# Supplementary material for: An invariant C-terminal tryptophan in McdB mediates its interaction and positioning function with carboxysomes
Source: Mol Biol Cell. 2024 Jul 11;35(8):ar107. doi: 10.1091/mbc.E23-11-0443 (PMC11321042; doi:10.1091/mbc.E23-11-0443)
Supplement: Supplementary file 1 [file mbc-35-ar107-s001.pdf]

Supplemental Materials

Molecular Biology of the Cell

Basalla *et al*.

Supplemental Figure S1: **McdB amino acid sequences are highly variable.** (**A**) A representative section of 50 sequences from the multiple sequence alignment of α-McdBs, centered on the sequence for *Hn* McdB (purple, starred). Dashes represent gaps in the alignment. (**B**) A representative section of 50 sequences from the multiple sequence alignment of β-McdBs, centered on the sequence for *Se* McdB (green, starred). Dashes represent gaps in the alignment. (**C**) A representative section of 50 sequences from the multiple sequence alignment of all McdBs, centered on a region in which α-McdBs (purple) and β-McdBs (green) align. Dashes represent gaps in the alignment. (**D**) Amino acid sequences of the full-length *Hn* and *Se* McdBs. The invariant tryptophan is colored green.

Supplemental Figure S2: **Removal of the invariant tryptophan of McdB results in carboxysome aggregation.** (**A**) Quantification of RbcS-mTQ intensities from carboxysome foci in the indicated strains of *Se*. Graphs show medians and interquartile ranges from 3 biological replicates each with n > 1000 foci. **** $p < 0.001$ from Mann-Whitney U-test. (**B**) As in (A), but for *Hn* strains. For exact cell and foci counts for each strain and replicate, see Supplemental Table S1.

Supplemental Figure S3: **Mutations to the invariant Trp does not affect McdB protein stability *in vivo* or *in vitro*.** (**A**) SEC-MALS for the indicated McdB proteins, with a summary table below. WT = wild type. (**B**) CD spectra for the indicated McdB proteins. (**C**) Quantification of mNG-McdB intensities per cell for the indicated strains. Graphs represent the medians and interquartile ranges for n > 500 cells for each strain. For exact cell and foci counts for each strain and replicate, see Supplemental Table S1.

Supplemental Figure S4: **The C-terminal domain of *Se* McdB alone does not oligomerize, but remains α-helical.** (**A**) SEC performed on the indicated column for the indicated protein variants. WT = wild type, NTD = N-terminal domain, CTD = C-terminal domain. Predicted monomeric weights are indicated for each construct. (**C**) CD spectra for the indicated proteins. Models of the constructs are shown, with wide cylinders representing α-helical regions and narrow cylinders representing region of intrinsic disorder.

Supplemental Figure S5: **Changing the invariant tryptophan to other aromatic residues reveals a gradient of McdB function in positioning carboxysomes.** (**A**) Quantification of RbcS-mTQ intensities from carboxysome foci in the indicated strains of *Se*. Graphs represent medians and interquartile ranges from 3 biological replicates each with n > 1000 foci. **** $p < 0.001$ from Mann-Whitney U-test. (**B**) As in (A), but for *Hn* strains. For exact cell and foci counts for each strain and replicate, see Supplemental Table S1.

Supplemental Figure S6: **Differences in the solubilities of *Se* McdB aromatic substitutions in *E. coli*.** (**A**) Representative microscopy image of the indicated *Se* mCherry-McdB variant after 3 hours of expression in *E. coli* MG1655. Phase contrast images are shown in black and white and overlaid with the fluorescence channel for mCherry-McdB as red. (**B**) Quantification of the proportion of cells from (A) with foci. Graphs represent means and standard deviations from 3 technical replicates with n = 993 cells for wild type, n = 2740 cells for W152Y, n = 1060 cells for W152F, and n = 1281 cells for ΔW152. *** $p < 0.001$, * $p < 0.05$, ns = non-significant from Welch's t-test. (**C**) (*top*) SDS-PAGE gel from the experiment shown in (A). Cells were harvested at the indicated times of expression and standardized by OD600 prior to running on the gel. Expected size of mCherry-McdB constructs from *Se* is roughly 45 kDa. (*bottom*) Quantification of the normalized band intensities from the above gel. Graph represents means and standard deviations from 3 technical replicates. Comparisons of all variants at the 3-hour time point were non-significant from Welch's t test. (**D**) Representative microscopy image of wild type *Hn* mNG-McdB after 3 hours of expression in *E. coli* BL21. Phase

contrast images are shown in black and white and overlaid with the fluorescence channel for mNG-McdB as yellow. Note that the protein remained completely soluble, and so mutant variants of *Hn* McdB were excluded from further analysis. Scale bars are 5 µm and apply to all images.

Supplemental Table 1: n values for individual replicates

Organism	Strain	Measurement	Replicate	n	Unit
S. elongatus	wild type	mTQ foci / micron	1	696	cells
S. elongatus	wild type	mTQ foci / micron	2	517	cells
S. elongatus	wild type	mTQ foci / micron	3	805	cells
S. elongatus	ΔW152	mTQ foci / micron	1	634	cells
S. elongatus	ΔW152	mTQ foci / micron	2	539	cells
S. elongatus	ΔW152	mTQ foci / micron	3	703	cells
S. elongatus	W152Y	mTQ foci / micron	1	648	cells
S. elongatus	W152Y	mTQ foci / micron	2	795	cells
S. elongatus	W152Y	mTQ foci / micron	3	539	cells
S. elongatus	W152F	mTQ foci / micron	1	653	cells
S. elongatus	W152F	mTQ foci / micron	2	512	cells
S. elongatus	W152F	mTQ foci / micron	3	528	cells
S. elongatus	wild type	mTQ foci intensity	1	1332	foci
S. elongatus	wild type	mTQ foci intensity	2	2429	foci
S. elongatus	wild type	mTQ foci intensity	3	1994	foci
S. elongatus	ΔW152	mTQ foci intensity	1	2119	foci
S. elongatus	ΔW152	mTQ foci intensity	2	2074	foci
S. elongatus	ΔW152	mTQ foci intensity	3	2050	foci
S. elongatus	W152Y	mTQ foci intensity	1	2335	foci
S. elongatus	W152Y	mTQ foci intensity	2	1793	foci
S. elongatus	W152Y	mTQ foci intensity	3	2220	foci
S. elongatus	W152F	mTQ foci intensity	1	2965	foci
S. elongatus	W152F	mTQ foci intensity	2	2235	foci
S. elongatus	W152F	mTQ foci intensity	3	2377	foci
S. elongatus	wild type	mNG mean intensity / cell	1	555	cells
S. elongatus	wild type	mNG mean intensity / cell	2	670	cells
S. elongatus	wild type	mNG mean intensity / cell	3	503	cells
S. elongatus	ΔW152	mNG mean intensity / cell	1	599	cells
S. elongatus	ΔW152	mNG mean intensity / cell	2	540	cells
S. elongatus	ΔW152	mNG mean intensity / cell	3	809	cells
H. neapolitanus	wild type	mTQ foci / micron	1	669	cells
H. neapolitanus	wild type	mTQ foci / micron	2	554	cells
H. neapolitanus	wild type	mTQ foci / micron	3	985	cells
H. neapolitanus	W94G	mTQ foci / micron	1	745	cells
H. neapolitanus	W94G	mTQ foci / micron	2	600	cells
H. neapolitanus	W94G	mTQ foci / micron	3	569	cells
H. neapolitanus	W94Y	mTQ foci / micron	1	746	cells
H. neapolitanus	W94Y	mTQ foci / micron	2	1179	cells
H. neapolitanus	W94Y	mTQ foci / micron	3	553	cells
H. neapolitanus	W94F	mTQ foci / micron	1	769	cells
H. neapolitanus	W94F	mTQ foci / micron	2	1211	cells
H. neapolitanus	W94F	mTQ foci / micron	3	739	cells
H. neapolitanus	wild type	mTQ foci intensity	1	745	foci
H. neapolitanus	wild type	mTQ foci intensity	2	1863	foci
H. neapolitanus	wild type	mTQ foci intensity	3	617	foci
H. neapolitanus	W94G	mTQ foci intensity	1	1636	foci
H. neapolitanus	W94G	mTQ foci intensity	2	662	foci
H. neapolitanus	W94G	mTQ foci intensity	3	641	foci
H. neapolitanus	W94Y	mTQ foci intensity	1	763	foci
H. neapolitanus	W94Y	mTQ foci intensity	2	562	foci
H. neapolitanus	W94Y	mTQ foci intensity	3	614	foci
H. neapolitanus	W94F	mTQ foci intensity	1	650	foci
H. neapolitanus	W94F	mTQ foci intensity	2	902	foci

H. neapolitanus	W94F	mTQ foci intensity	3	997	foci

Supplemental Table S2: strains used in this study.

Strain #	Organism	McdB Variant	Genotype	Source
C28	*S. elongatus*	mNG-wild type	ΔmcdA::mNG-mcdA (KmR), NS1::RbcS-mTQ (CmR)	Ref 10
C50	*S. elongatus*	mNG-ΔW152		This Study
C52	*S. elongatus*	mNG-W152Y		This Study
C51	*S. elongatus*	mNG-W152F		This Study
C99	*S. elongatus*	mNG-CTD		This Study
Hn510	*H. neapolitanus*	mNG-wild type		This Study
Hn513	*H. neapolitanus*	mNG-W94G		This Study
Hn511	*H. neapolitanus*	mNG-W94Y		This Study
Hn512	*H. neapolitanus*	mNG-W94F		This Study
Hn506	*H. neapolitanus*	mNG-CTD		This Study
Hn2	*H. neapolitanus*	wild type		Ref 23
Hn516	*H. neapolitanus*	W94G		This Study
Hn514	*H. neapolitanus*	W94Y		This Study
Hn515	*H. neapolitanus*	W94F		This Study

Supplemental Table S2: strains used in this study.

Strain #	Organism	Genotype	Source
C28	*S. elongatus*	*ΔmcdB::mNG-mcdB (KmR), NS1::RbcS-mTQ (CmR)*	Ref 10
C50	*S. elongatus*	*ΔmcdB::mNG-mcdB[ΔW152] (KmR), NS1::RbcS-mTQ (CmR)*	This Study
C52	*S. elongatus*	*ΔmcdB::mNG-mcdB[W152Y] (KmR), NS1::RbcS-mTQ (CmR)*	This Study
C51	*S. elongatus*	*ΔmcdB::mNG-mcdB[W152F] (KmR), NS1::RbcS-mTQ (CmR)*	This Study
C99	*S. elongatus*	*ΔmcdB::mNG-mcdB[CTD] (KmR), NS1::RbcS-mTQ (CmR)*	This Study
Hn510	*H. neapolitanus*	*ΔmcdB::mNG-mcdB (CmR), NS1::Cbbs-mTQ (KmR)*	This Study
Hn513	*H. neapolitanus*	*ΔmcdB::mNG-mcdB[W94G] (CmR), NS1::Cbbs-mTQ (KmR)*	This Study
Hn511	*H. neapolitanus*	*ΔmcdB::mNG-mcdB[W94Y] (CmR), NS1::Cbbs-mTQ (KmR)*	This Study
Hn512	*H. neapolitanus*	*ΔmcdB::mNG-mcdB[W94F] (CmR), NS1::Cbbs-mTQ (KmR)*	This Study
Hn506	*H. neapolitanus*	*ΔmcdB::mNG-mcdB[CTD10] (CmR), NS1::Cbbs-mTQ (KmR)*	This Study
Hn2	*H. neapolitanus*	*NS1::Cbbs-mTQ (KmR)*	Ref 23
Hn516	*H. neapolitanus*	*ΔmcdB::mcdB[W94G] (CmR), NS1::Cbbs-mTQ (KmR)*	This Study
Hn514	*H. neapolitanus*	*ΔmcdB::mcdB[W94Y] (CmR), NS1::Cbbs-mTQ (KmR)*	This Study
Hn515	*H. neapolitanus*	*ΔmcdB::mcdB[W94F] (CmR), NS1::Cbbs-mTQ (KmR)*	This Study

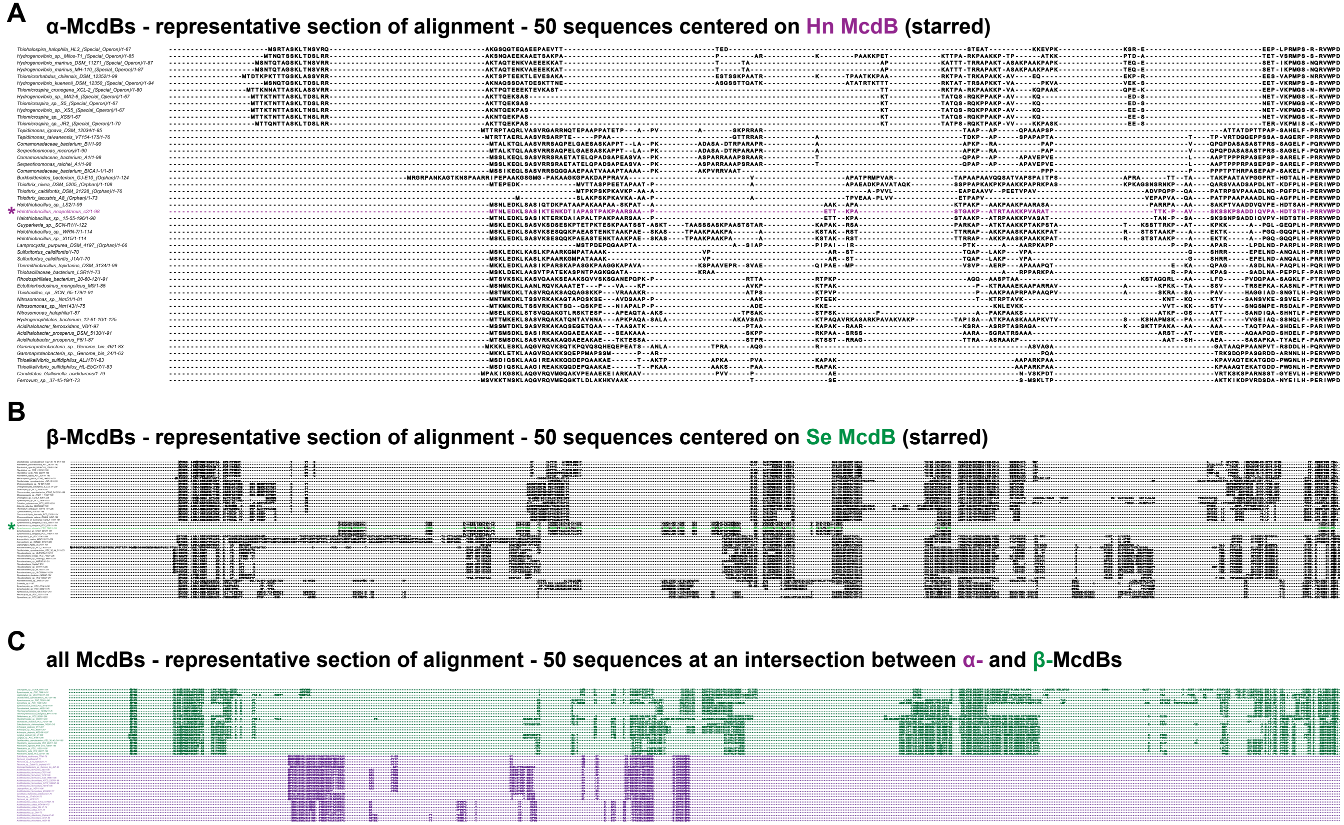

D

Hn McdB:

```
MTNLEDKLSA SIKTENKDTI APASTPAKPA ARSAAPETTK PASTGAKPAT
RTAAKKPVAR ATTTKPAVSK SSKPSADDIQ VPAHDTSTHP RRVWPD
```

Se McdB:

```
MTDAFDRLKK RSRTPIAREG SLTTGPELSD RPLQLLPREF ETFCDRYAVH
AGDVIEAALD LVLLDPDLQQ RLLQRLRQGN GSDRVWLGTA CPRSWQQQLQ
QQAQDQGLSE ADLLQEAIAQ RLDLVLGQTT LREEVTLLRQ ELDQLKRKLH GW
```

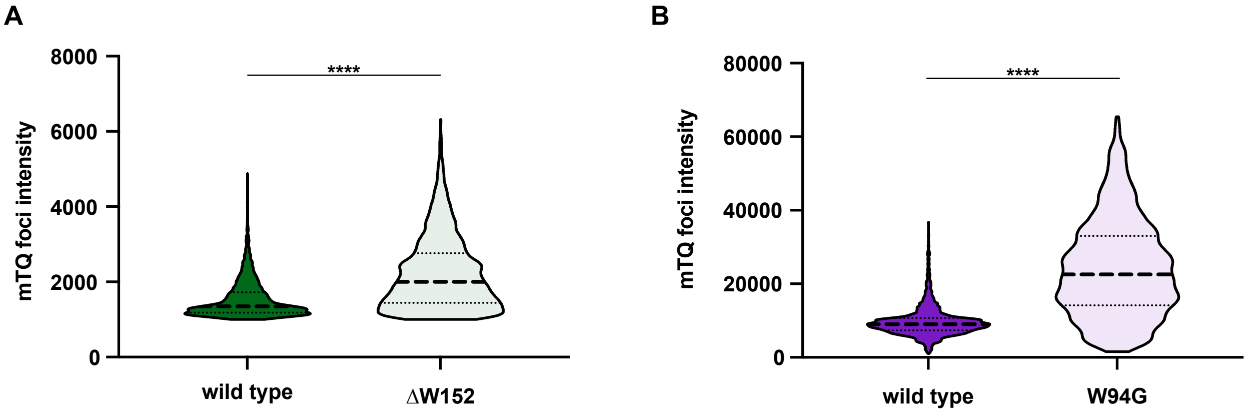
A
B
mTQ foci intensity
8000
6000
4000
2000
0
****
wild type
ΔW152
80000
60000
40000
20000
0
W94G

A

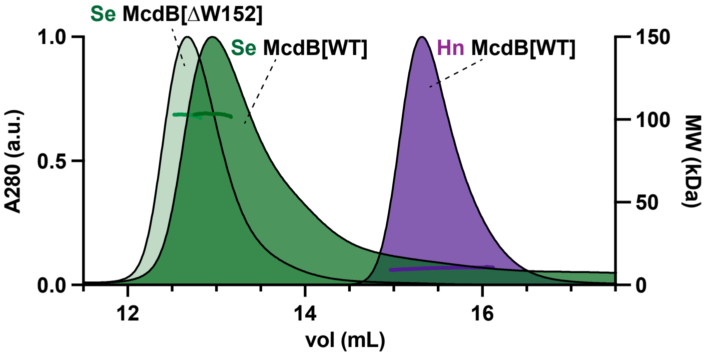

	monomer (kDa)	oligomer (kDa)	multiple	oligomer
Se McdB[WT]	17.4	103.2	x5.9	hexamer
Se McdB[ΔW152]	17.2	102.6	x6.0	hexamer
Hn McdB[WT]	10.0	10.1	x1.0	monomer

B

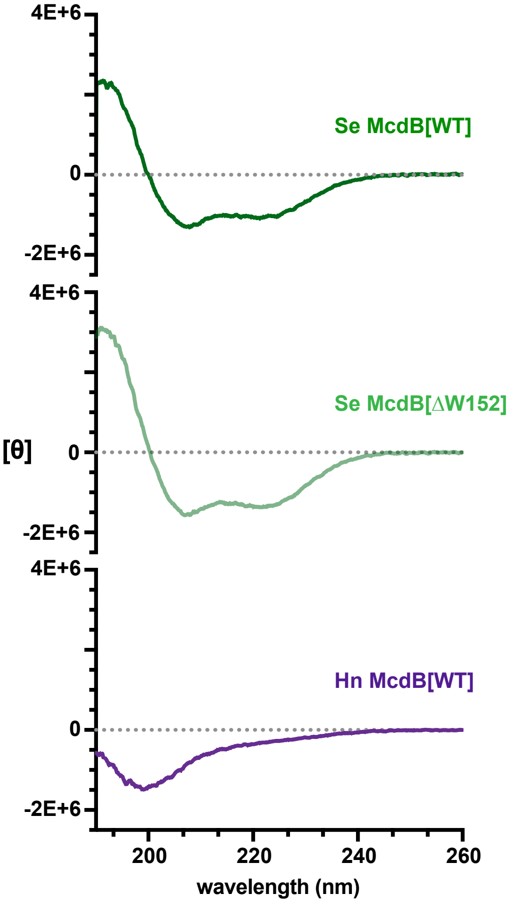

C

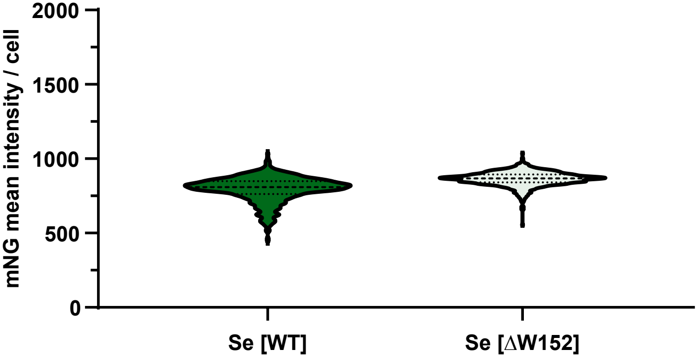

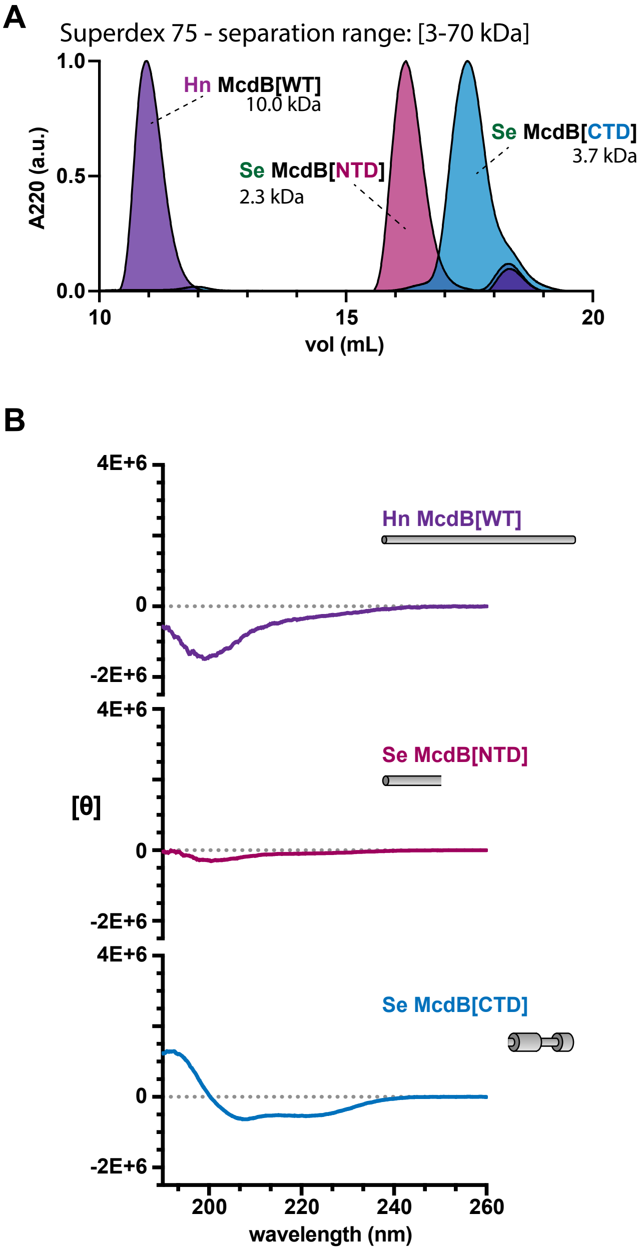

A
Superdex 75 - separation range: [3-70 kDa]
Hn McdB[WT]
10.0 kDa
Se McdB[CTD]
3.7 kDa
Se McdB[NTD]
2.3 kDa
A220 (a.u.)
1.0
0.5
0.0
10
15
20
vol (mL)
B
Hn McdB[WT]
Se McdB[NTD]
Se McdB[CTD]
[θ]
4E+6
0
-2E+6
200
220
240
260
wavelength (nm)

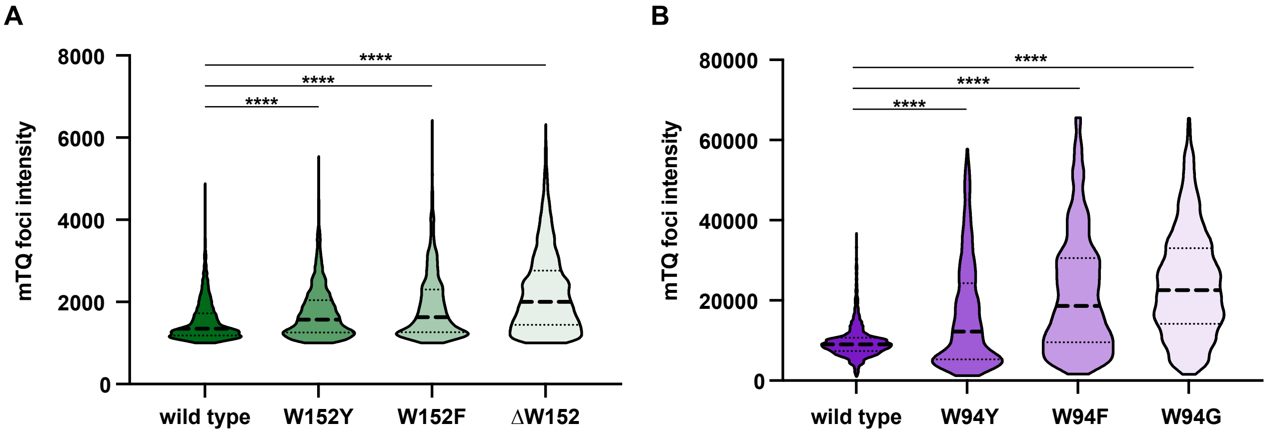
A
mTQ foci intensity
8000
6000
4000
2000
0
****
****
****
wild type
W152Y
W152F
ΔW152
B
mTQ foci intensity
80000
60000
40000
20000
0
****
****
****
wild type
W94Y
W94F
W94G

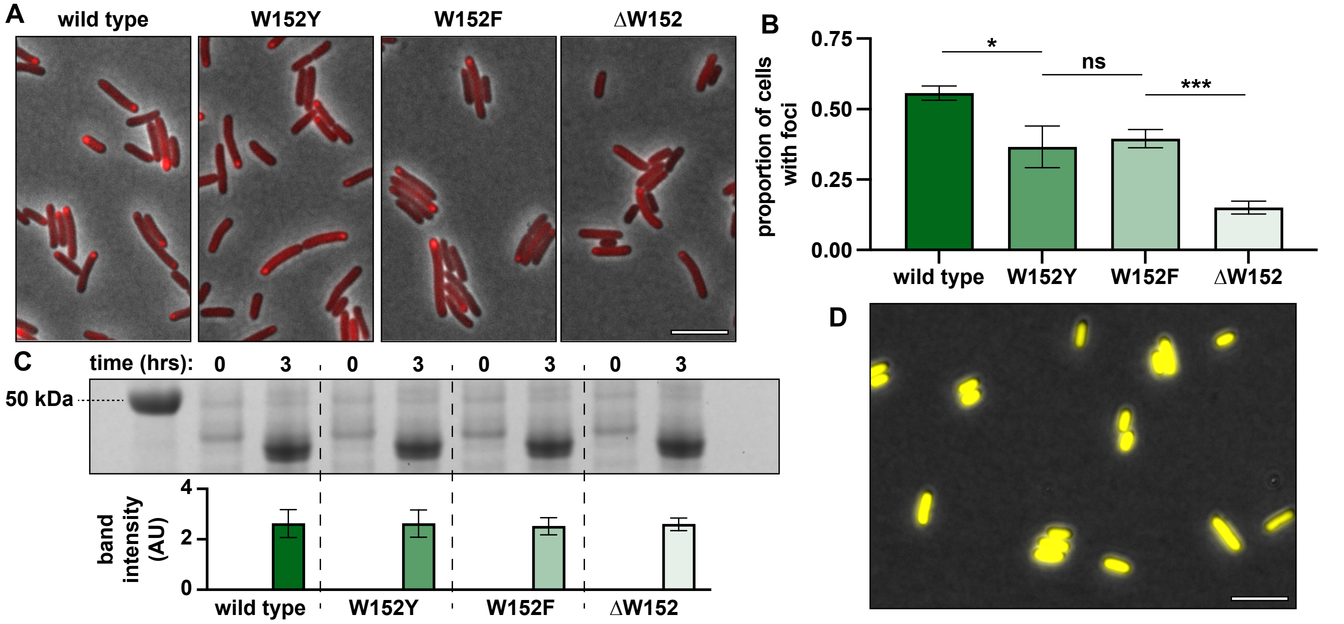
A
wild type
W152Y
W152F
ΔW152
B
0.75
0.50
0.25
0.00
proportion of cells with foci
*
ns
***
wild type
W152Y
W152F
ΔW152
C
time (hrs):
0
3
0
3
0
3
0
3
50 kDa
4
2
0
band intensity (AU)
wild type
W152Y
W152F
ΔW152
D
