# Supplementary material for: An invariant C-terminal tryptophan in McdB mediates its interaction and positioning function with carboxysomes
Source: Mol Biol Cell. 2024 Jul 11;35(8):ar107. doi: 10.1091/mbc.E23-11-0443 (PMC11321042; doi:10.1091/mbc.E23-11-0443)
Supplement: Supplementary file 2 [file reviewer_reportE23-11-0443.pdf]

An invariant C-terminal tryptophan in McdB mediates its interaction and positioning function with carboxysomes

Joseph Basalla, Maria Ghalmi, Hoang Y, Rachel Dow, and Anthony Vecchiarelli

Corresponding author(s): Anthony Vecchiarelli, University of Michigan

**Review Timeline:**		
	Submission Date:	2023-11-20
	Editorial Decision:	2023-12-12
	Revision Received:	2024-06-10
	Accepted:	2024-06-11

Editor-in-Chief: Matthew Welch

Transaction Report:

(Note: With the exception of the correction of typographical or spelling errors that could be a source of ambiguity, letters and reports are not edited. The original formatting of letters and referee reports may not be reflected in this compilation.)

RE: Manuscript #E23-11-0443
TITLE: An invariant C-terminal tryptophan in McdB mediates its interaction and positioning function with carboxysomes

Dear Dr. Vecchiarelli:

Thanks for your submission to MBoC. You'll see that the two reviewers both agreed that your manuscript is a valuable contribution to our understanding of carboxysome positioning with broader relevance to cellular organization. Each reviewer suggests points to consider to further strengthen the manuscript, which should be addressed in full in a revision. With respect to additional experiments, please consider in particular point 1 from reviewer 1 to include western blots to ensure fusion proteins are intact. In addition, please double check the MBoC Author Submission Checklist for compliance (for example, please provide exact n for each experiment, not a range).

Sincerely,

Erin Goley
Monitoring Editor
Molecular Biology of the Cell

Dear Dr. Vecchiarelli,

The review of your manuscript, referenced above, is now complete. The Monitoring Editor has decided that your manuscript is not acceptable for publication at this time, but may be deemed acceptable after specific revisions are made, as described in the Monitoring Editor's decision letter above and the reviewer comments below.

A reminder: Please do not contact the Monitoring Editor directly regarding your manuscript. If you have any questions regarding the review process or the decision, please contact the MBoC Editorial Office (mboc@ascb.org).

When submitting your revision include a rebuttal letter that details, point-by-point, how the Monitoring Editor's and reviewers' comments have been addressed. (The file type for this letter must be "rebuttal letter"; do not include your response to the Monitoring Editor and reviewers in a "cover letter.") Please bear in mind that your rebuttal letter will be published with your paper if it is accepted, unless you have opted out of publishing the review history.

Authors are allowed 90 days to submit a revision. If this time period is inadequate, please contact us at mboc@ascb.org.

Revised manuscripts are assigned to the original Monitoring Editor whenever possible. However, special circumstances may preclude this. Also, revised manuscripts are often sent out for re-review, usually to the original reviewers when possible. The Monitoring Editor may solicit additional reviews if it is deemed necessary to render a completely informed decision.

In preparing your revised manuscript, please follow the instruction in the Information for Authors (www.molbiolcell.org/info-for-authors). In particular, to prepare for the possible acceptance of your revised manuscript, submit final, publication-quality figures with your revision as described.

To submit the rebuttal letter, revised manuscript, and figures, use this link: Link Not Available

MBoC offers the option to publish your work with immediate open access. Open access can increase the discoverability and usability of your research. If you would like to publish your paper with immediate open access but did not select this option during initial submission, please contact the editorial office at mbc@ascb.org.

Thank you for submitting your manuscript to MBoC. We look forward to receiving your revised paper.

Sincerely,

Eric Baker
Journal Production Manager
MBoC Editorial Office
mbc@ascb.org

Reviewer #1 (Remarks to the Author):

This paper reports the identification of a conserved interaction determinant that mediates the association of McdB-like carboxysome positioning proteins with α- and β-carboxysomes. Using multiple sequence alignments, the authors show that McdB proteins consistently contain a tryptophan residue at or close to the C-terminus. In vivo studies then reveal that variants lacking this residue no longer colocalize with carboxysomes and fail to mediate carboxysome positioning. In vitro studies suggest that the functional defects are not caused by protein misfolding or degradation. The C-terminal 10 amino acids of the Hn McdB are sufficient for binding to carboxysomes, whereas the terminal 31 amino acids of Se McdB are not, which is explained by a difference in the oligomerization state of the full-length proteins. McdB variants in which the conserved tryptophan residues were replaced by other aromatic residues showed a gradient of functionality in vivo that correlated with a gradient in the ability to undergo liquid-liquid phase separation in vitro. Based on these findings the authors speculate that phase separation may be relevant to the function of McdB.

Overall, this is an interesting paper showing the existence of a common interaction determinant in evolutionarily distinct McdAB carboxysome positioning systems. The study is well-planned, the data are of high quality and the writing is clear. However, there are a few issues that should be addressed before publication:

1. The authors use in vitro analyses of native proteins and an in vivo quantification of mNG fluorescence as evidence that the mNG-McdB fusions are stable. However, these assays cannot exclude the possibility that the diffuse signal observed for mutant fusion proteins is due to cleavage of the fluorescence tag rather than a reduced affinity for carboxysomes. The results obtained for the mCherry fusions in E. coli are not suitable to conclude on the stability of the fusions in other species. Therefore, Western blot analyses should be performed with the proteins analyzed in Figures 2, 4 and 5.

2. AlphaFold2 models of the two McdB proteins investigated should be included, in particular to assess whether the 31 aa Se McdB fragment is an independent domain or an integral part of a folded protein.

3. The authors correlate the results of in vitro phase separation assays performed with native proteins to those of in vivo localization studies performed with fusion proteins. Is it clear that the fusion proteins show the same (or at least a similar) tendency to undergo liquid-liquid phase separation as the non-tagged proteins?

4. In their quantitative analyses, the authors state that the graphs show the means and SD of several biological/technical replicates. Does this mean they determined the means of each replicate and then determined the mean and SD of these mean values, or were all the replicates combined into a single dataset? Please clarify. In principle, it would be better to represent quantitative imaging data as SuperPlots (Lord et al, J Cell Biol, 2020) to provide a more realistic measure of the biological/technical variability of the data.

5. The calculated proportions of α-helices, β-sheets and non-structured regions should be provided for CD spectra.

Other comments/suggestions:

- throughout: In many cases, the special character "ß" is used instead of the Greek letter "β".

- line 13: Perhaps clearer: "gradients in the efficiency of"

- line 114ff: "proteins contain" instead "proteins encode"

- throughout: In many cases, the expression "C-terminal region" would be more appropriate than "C-terminus", which refers to the very C-terminal end of a peptide chain.

- The noun "mutant" should only be used when referring to a mutant organism, not to a protein.

- line 167: Perhaps clearer: "the fusion to mNG destroys the ... of McdB"

- line 219: The data do not show that the 10 amino acids are "necessary". Only the Trp residue was shown to be necessary. It is unclear whether this peptide could be further shortened without any loss in activity.

- line 221: "are sufficient"

- The authors strongly focus on the effect of the different amino exchanges on condensate formation as an explanation of the gradient in functionality they observe. Another (additional) explanation may be that the conserved Trp residue inserts into a hydrophobic pocket in the interacting protein and this interaction is weakened if the hydrophobic character of the amino acid decreases.

- Can the authors speculate on how McdB condensate formation could aid in the positioning of carboxysomes? Would a key role of the conserved Trp residues in condensate formation exclude a stable (or quantitative) interaction of this residue with the target protein?

- line 427ff: The study proposing the condensate-like nature of ParB partition complexes (ref. 46) did not consider recent key advances in the understanding of ParB function when interpreting the data obtained. Its findings should be evaluated in the context of current knowledge and perhaps not be presented as strong evidence of a condensate-based mechanism of ParB function in vivo.

- line 429: In the PomXYZ system, the PomY condensate appears to be the target, rather than the adapter of the PomZ-based positioning system. The adapter function is exerted by PomX, whose N-terminal region mediates the interaction of the PomXY complex with PomZ (Schumacher et al, eLife, 2021).

- line 561: "Circular dichroism spectroscopy"

- legend to Figure S4: "(B)" instead of "(C)"

Reviewer #2 (Remarks to the Author):

The study by Basalla and colleagues provides data to support the role of a tryptophan residue within the C-terminus of McdB that plays a role in co-localizing McdB to carboxysomes. In addition, they also established a clear correlation in which the same substitutions of this C-terminal tryptophan reduced the ability of McdB to form condensates in vitro. The study provides rigorous data that is analyzed quantitatively, demonstrating the importance of the C-terminal tryptophan to McdB's subcellular localization, function, and condensate formation in vitro. Based on these findings, the authors propose a model in which McdB condensate formation is critical for the association with carboxysomes. In addition, the authors provided an informative discussion that placed their work in a broader context, making links to Kinesin-1 and viral capsid assembly. Overall, it is interesting as it may suggest that the static carboxysome shells have a liquid-like McdB coating, which is critical for carboxysome partitioning. This study would be of broad interest to those who study carboxysome and bacterial cell biology.

I encourage the authors to consider the following, which may help improve their study:

(1) A minor weakness is that the manuscript utilizes a single type of experiment to demonstrate the C-terminal residues and tryptophan are critical for carboxysome association (i.e., colocalization with the carboxysome in vivo). The authors could consider leveraging the bacterial two-hybrid assay used in their lab's work in Ref 10 to add an additional line of evidence. Ref 10 utilized the bacterial two-hybrid assay to provide evidence for McdB's interaction with CcmK2, CcmK3, CcmK4, CcmL, and CcmO. Using this assay and existing controls, the authors could demonstrate a loss of interaction with CcmK2, CcmK3, CcmK4, CcmL, or CcmO with the studied McdB variants. If performed this would provide two independent types of experiment that collectively point to the role of the C-terminus in carboxysome association.

(2) Studies with the Se CTD demonstrated a lack of carboxysome association and also reduced phase separation in their lab's previous study (Basalla, Elife 2023). This suggests that the McdB association with carboxysomes is linked with phase separation. HnCTD may diverge from this observation (Fig 3D). In comparison, HnCTD was sufficient for carboxysome association in vivo (Fig 3D). This raises the question, does HnCTD also phase separate in vitro (unlike the SeCTD variant)? If Hn CTD does not phase separate, this could provide an example of McdB association without phase separation. Due to the 10-mer's small size, it could be obtained by peptide synthesis if protein expression/purification was challenging. It's also possible this was analyzed in past work and could be referenced and discussed.

(3) In the abstract, the authors implicate condensate formation as pivotal in associating with carboxysomes in vivo. This conclusion is based on a solid correlation between the in vivo colocalization data and in vitro phase separation. Interestingly, this idea further suggests that McdB may form a dynamic liquid-like coating on the carboxysome surface versus a more static coating that one would expect from a typical carboxysome shell protein. Related to this observation, a few questions:

a. For a protein like McdB to work, could the authors elaborate/speculate the functional significance of associating in a way that leverages phase separation versus just being a more static shell protein of the carboxysome?

b. There are many examples of phase separation constrained in two dimensions by membrane association. Do you think that McdB phase separation is similarly constrained in two dimensions by the protein shell of the carboxysome? Or does McdB form more 3-dimensional liquid-like droplets on the surface of condensates? Are there functional advantages to either state? Are there other examples in the literature where phase separation is contained in 2 dimensions by a more solid-like protein shell or protein filament?

The following two suggestions intend to help strengthen the paper's conclusions but may take more work and could be considered in a more in-depth follow-up study.

c. To strengthen this paper's conclusion about in vivo McdB phase separation, it would be interesting to see the FRAP of mNG-McdB versus a more static shell protein or the entrapped rubisco protein (RbsC-mTQ). If a difference were observed, this would provide compelling in vivo evidence that phase separation was involved in carboxysome association and that McdB is not just a static carboxysome shell adaptor protein.

d. To demonstrate the importance of phase separation in this association process, it would be nice to have a McdB variant that does not phase separate but binds the carboxysome shell (e.g. by inclusion of a solubilization tag such as MBP). This, however, may be a bit tricky to identify and validate.

Reviewer #1 (Remarks to the Author):

Major Points:

1. The authors use in vitro analyses of native proteins and an in vivo quantification of mNG fluorescence as evidence that the mNG-McdB fusions are stable. However, these assays cannot exclude the possibility that the diffuse signal observed for mutant fusion proteins is due to cleavage of the fluorescence tag rather than a reduced affinity for carboxysomes. The results obtained for the mCherry fusions in E. coli are not suitable to conclude on the stability of the fusions in other species. Therefore, **Western blot analyses** should be performed with the proteins analyzed in Figures 2, 4 and 5.

   Thank you, this is an excellent point. Although the suggestion is unlikely, without Western Blots, we cannot formally rule out the possibility that the C-terminal Trp mutations are not somehow causing N-terminal mNG-tag cleavage and McdB instability *in vivo.* We did try to address this using Western Blots, but had difficulty getting reliable quantitative signals using α-mNG and α-McdB antibodies. However, as noted by the reviewer, we address this using several indirect measures:

   1. Deletion of the invariant tryptophan does not cause decreased mNG levels *in vivo.*
   2. All McdB variants were reproducibly expressed and purified from *E. coli* with no statistically significant differences in expression level or construct stability.
   3. Deletion of the invariant tryptophan showed the same biochemical signatures (CD, SEC-MALS) as wild type McdB.

   We understand that, although unlikely, the issue is still a formal possibility, but only for the McdB Trp mutants that phenocopy an McdB deletion. It is important to note that the majority of our Trp mutants do not phenocopy an McdB deletion mutant in regards to carboxysome mispositioning; specifically the W → F and W → Y substitutions. Furthermore, W → Y even restored WT levels of carboxysome colocalization in *S. elongatus*. Although we did not originally state this in the text, this data strongly supports the fact that these mutations to the C-terminal invariant tryptophan do not somehow lead to the N-terminal mNG tag being cleaved, with only the McdB sequence being somehow specifically degraded. Rather, the more parsimonious explanation on the entire mutant data set is that there is a specific effect on McdB-carboxysome affinity and carboxysome positioning function.

   Ultimately, the reviewer raises a formal possibility that specifically pertains to only our Trp mutants that phenocopy an McdB deletion mutant. Therefore, we have now added text to the manuscript at **lines 268-271** explicitly mentioning this caveat for those specific mutations of the invariant Trp (DeltaTrp in Se, W → G in Hn).

2. **AlphaFold2 models** of the two McdB proteins investigated should be included, in particular to assess whether the 31 aa Se McdB fragment is an independent domain or an integral part of a folded protein.

   Thank you for this comment. We performed several assays in our previous study that strongly support the proposal that the CTD of Se McdB is a stable, independent domain (Basalla et al., 2023, eLife). This study is cited in the relevant section, **line 210**.

   Additionally, we have performed AlphaFold 2 modelling of McdB, but it does not produce reliable structures. This is in largely due to McdB having low sequence identity with all sequences in the PDB. For example, see the I-TASSER results from our previous study (Basalla et al., 2023, eLife), showing very low sequence identity of McdB versus the PDB.

3. The authors correlate the results of in vitro phase separation assays performed with native proteins to those of in vivo localization studies performed with **fusion proteins**. Is it clear that the fusion proteins show the same (or at least a similar) tendency to undergo **liquid-liquid phase separation** as the non-tagged proteins?

Excellent point. As other groups have observed (e.g. see Pandey et. al 2024, JBC), fluorescent tags can certainly shift the phase boundaries for protein condensate formation. However, the tags do not completely abolish the ability of these proteins to form condensates, nor do they completely change the chemistry of the tagged protein. Therefore, even though the mNG tag may affect the degree of McdB solubility, the mutations made to the invariant tryptophan will have the same relative effect on McdB condensate formation whether or not the protein is tagged, and thus the *in vitro* results of untagged McdB are still relevant to the behavior of the tagged McdB variants *in vivo*. This is evident by the data in Supp. Fig. 6 which shows that mCherry-McdB variants show the same trend in solubility as the untagged versions *in vitro*. However, to the reviewer's point, we now mention this caveat on **lines 318-319**.

4. In their quantitative analyses, the authors state that the graphs show the means and SD of several biological/technical replicates. Does this mean they determined the means of each replicate and then determined the **mean and SD** of these mean values, or were all the replicates combined into a single dataset? Please clarify. In principle, it would be better to represent quantitative imaging data as SuperPlots (Lord et al, J Cell Biol, 2020) to provide a more realistic measure of the biological/technical variability of the data.

Thank you, each replicate was kept separate, and the reported mean was determined from n number of means. Therefore, n was the number of replicates, not number of cells, as recommended by Lord et al. We have updated the relevant methods section to clarify our approach.

And thank you for your suggestion. We will be sure to keep SuperPlots in mind for future studies. As a reference, see the representative plots below which show that the variation between replicates was minor.

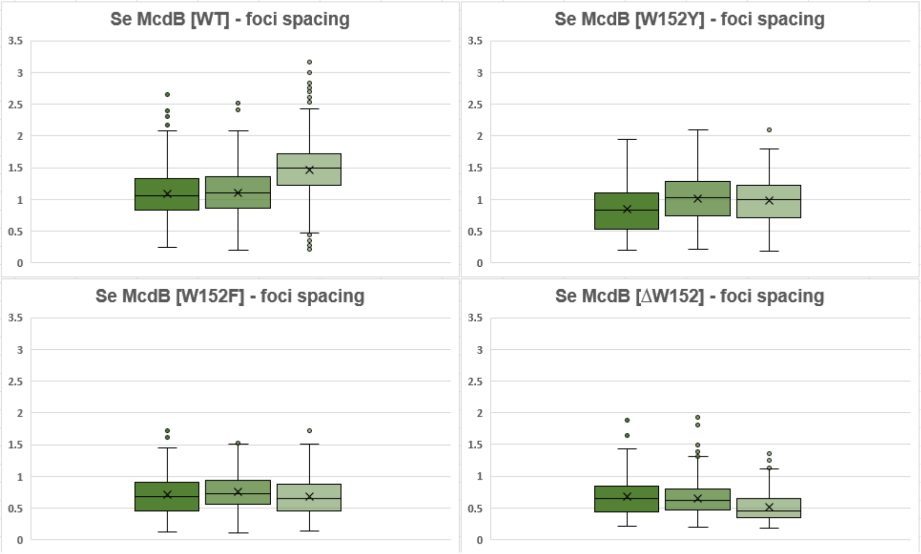

5. The calculated **<u>proportions of α-helices, β-sheets</u>** and non-structured regions should be provided for CD spectra.

   We agree that this type of analysis can provide better resolution to CD data. However, we would like to note that we used CD in two supplemental figures as evidence to support our claims as described below, and higher resolution to this data would not further strengthen our specific claims made here. For instance:

   - In Figure S3, CD was used to supplement the supplementary SEC-MALS data to show that there was no gross change to McdB structure. If SEC-MALS showed differences in oligomerization, then a more detailed CD analysis may have been helpful to determine why. But this wasn't the case.

   - In Figure S4, CD was used to supplement the supplementary SEC data to show that Se McdB CTD is overall less disordered than Se McdB NTD. The data show clear disordered versus alpha-helical signatures, which are sufficient to make our claim.

Minor Points:

1. throughout: In many cases, the special character "ß" is used instead of the Greek letter "β".

   Thank you, this has been corrected.

2. line 13: Perhaps clearer: "gradients in the efficiency of"

   Thank you, this has been corrected.

3. line 114ff: "proteins contain" instead "proteins encode"

   Thank you, this has been corrected.

4. throughout: In many cases, the expression "C-terminal region" would be more appropriate than "C-terminus", which refers to the very C-terminal end of a peptide chain.

   Thank you, this has been corrected.

5. The noun "mutant" should only be used when referring to a mutant organism, not to a protein.

   Thank you, we have tried to reword when necessary to ensure clarity that we are referencing a mutated version of the protein and not the associated organism.

6. line 167: Perhaps clearer: "the fusion to mNG destroys the ... of McdB"

   Thank you, this has been corrected.

7. line 219: The data do not show that the 10 amino acids are "necessary". Only the Trp residue was shown to be necessary. It is unclear whether this peptide could be further shortened without any loss in activity.

   Thank you, this has been corrected.

8. line 221: "are insufficient"

   Thank you, this has been corrected.

9. The authors strongly focus on the effect of the different amino exchanges on condensate formation as an explanation of the gradient in functionality they observe. Another (additional) explanation may be that the conserved Trp residue inserts into a **hydrophobic pocket** in the interacting protein and this interaction is weakened if the hydrophobic character of the amino acid decreases.

   Thank you, this is a good point. We agree that condensate formation can only partially explain McdB localization to carboxysomes, and that additional interactions, potentially with a hydrophobic pocket, likely also play a role. However, we would like to note that the gradient in colocalization we saw was structured as Trp > Tyr > Phe. Notably, tyrosine is *less* hydrophobic than phenylalanine, and so decreases in McdB localization to carboxysomes do not completely correlate with decreases in hydrophobicity. Therefore, we propose condensate formation (which does correlate with McdB localization) may be playing a relevant role. However, to the reviewers point, the role of a hydrophobic pocket is certainly worth discussing, and we have added text starting on **line 371** to address this.

10. Can the authors speculate on how McdB condensate formation could aid in the positioning of carboxysomes? Would a key role of the conserved Trp residues in condensate formation exclude a stable (or quantitative) interaction of this residue with the target protein?

    Thank you. We have tried to integrate our response to this with our response to the reviewer's previous comment in a new discussion section beginning on **line 356**.

11. line 427ff: The study proposing the condensate-like nature of ParB partition complexes (ref. 46) did not consider recent key advances in the understanding of ParB function when interpreting the data obtained. Its findings should be evaluated in the context of current knowledge and perhaps not be presented as strong evidence of a condensate-based mechanism of ParB function in vivo.

    Thank you and we agree. There is currently limited data supporting the conclusion that ParB forms a liquid-like condensate. But it is for this reason, we were very intentional in our wording. Even prior to the more recent works highlighted by the reviewer regarding the function of ParB, several lines of evidence point strongly against the proposal of liquid-liquid phase separation (LLPS). However, we were intentional here to use terminology that does not infer assembly mechanisms (ie LLPS) or material state of the complex. ParB loading onto and around parS in a manner regulated by its CTPase cycle can still be described as phase separation and the complex can still be described as a biomolecular condensate, as these terms do not infer an assembly mechanism or material state of the complex. For example, the CTP-regulated interactions of ParB with itself, with parS, and surrounding non-specific DNA can still be modeled as phase separation coupled to percolation (PSCP) or potentially other

forms of phase transition (see Mittag and Pappu, Mol Cell 2022). But as the reviewer points out, the data in the field strongly suggests that it is not LLPS. We believe these nuanced discussions of cargo assembly mechanisms require a dedicated review or perspective piece. Therefore, with the addition of our new discussion section, we felt the discussion as a whole was getting lengthy. So the section regarding adaptor proteins has been removed as we thought it was more productive to focus on McdB in the context of carboxysomes instead of the context of ParA/MinD-family ATPases.

12. line 429: In the PomXYZ system, the PomY condensate appears to be the target, rather than the adapter of the PomZ-based positioning system. The adapter function is exerted by PomX, whose N-terminal region mediates the interaction of the PomXY complex with PomZ (Schumacher et al, eLife, 2021).

    Thank you. Please see our response to comment 11.

13. line 561: "Circular dichroism spectroscopy"

    Thank you, this has been corrected.

14. legend to Figure S4: "(B)" instead of "(C)"

    Thank you, this has been corrected.

Reviewer #2 (Remarks to the Author):

15. A minor weakness is that the manuscript utilizes a single type of experiment to demonstrate the C-terminal residues and tryptophan are critical for carboxysome association (i.e., colocalization with the carboxysome in vivo). The authors could consider leveraging the **bacterial two-hybrid assay** used in their lab's work in Ref 10 to add an additional line of evidence. Ref 10 utilized the bacterial two-hybrid assay to provide evidence for McdB's interaction with CcmK2, CcmK3, CcmK4, CcmL, and CcmO. Using this assay and existing controls, the authors could demonstrate a loss of interaction with CcmK2, CcmK3, CcmK4, CcmL, or CcmO with the studied McdB variants. If performed this would provide two independent types of experiment that collectively point to the role of the C-terminus in carboxysome association.

    Thank you, this is a good point. We did attempt to perform B2H assays on these mutants, but had difficulty with the assay. One caveat of the B2H in this context is that McdB could interact with any surface of the isolated shell protein, which is markedly different than how McdB interacts with fully assembled and functional carboxysomes in vivo. To the reviewer's point, we have tried to refrain from making strong claims on McdB-carboxysome interaction, and use terminology such as "colocalization" instead of "interaction" in the text. How McdB associates with the multi-component shell and core of a carboxysome is a very important question we hope to address in a future study.

16. Studies with the Se CTD demonstrated a lack of carboxysome association and also reduced phase separation in their lab's previous study (Basalla, Elife 2023). This suggests that the McdB association with carboxysomes is linked with phase separation. HnCTD may diverge from this observation (Fig 3D). In comparison, HnCTD was

sufficient for carboxysome association in vivo (Fig 3D). This raises the question, **does HnCTD also phase separate in vitro** (unlike the SeCTD variant)? If Hn CTD does not phase separate, this could provide an example of McdB association without phase separation. Due to the 10-mer's small size, it could be obtained by peptide synthesis if protein expression/purification was challenging. It's also possible this was analyzed in past work and could be referenced and discussed.

Thank you, this is a very good point. Efforts are currently ongoing to systematically investigate the grammar of Hn McdB condensate formation. In congruence with questions raised by another reviewer, we have added a discussion section starting on **line 356** to the text and have included this point as a future direction.

17. In the abstract, the authors implicate condensate formation as pivotal in associating with carboxysomes in vivo. This conclusion is based on a solid correlation between the in vivo colocalization data and in vitro phase separation. Interestingly, this idea further suggests that McdB may form a dynamic liquid-like coating on the carboxysome surface versus a more static coating that one would expect from a typical carboxysome shell protein. Related to this observation, a few questions:

    a. For a protein like McdB to work, could the authors elaborate/speculate the **functional significance of associating in a way that leverages phase separation** versus just being a more static shell protein of the carboxysome?

       Good point. As mentioned above, we have added a discussion section focused on the potential role of McdB phase separation and have discussed some of the biological context surrounding our model.

    b. There are many examples of **phase separation constrained in two dimensions** by membrane association. Do you think that McdB phase separation is similarly constrained in two dimensions by the protein shell of the carboxysome? Or does McdB form more 3-dimensional liquid-like droplets on the surface of condensates? Are there functional advantages to either state? Are there other examples in the literature where phase separation is contained in 2 dimensions by a more solid-like protein shell or protein filament?

       Good point. There is excellent work by other labs discussing the role of dimensionality in phase separation; namely "surface-assisted" condensation. We have added this to the new discussion section.

18. The following two suggestions intend to help strengthen the paper's conclusions but may take more work and could be considered in a more in-depth **follow-up study**.

    c. To strengthen this paper's conclusion about in vivo McdB phase separation, it would be interesting to see the **FRAP of mNG-McdB versus a more static shell protein** or the entrapped rubisco protein (RbsC-mTQ). If a difference were observed, this would provide compelling in vivo evidence that phase separation was involved in carboxysome association and that McdB is not just a static carboxysome shell adaptor protein.

       Thank you, this is an excellent suggestion. There are a few technical difficulties to these experiments that would likely make this more useful for a follow-up study, but is certainly worth pursuing. Some of the major shell proteins are not amenable to fluorescent tagging, making this approach difficult.

Furthermore, the dynamics of the shell, and whether this changes under different conditions, is not well known and so preliminary data on shell dynamics alone would be useful before investigating McdB. However, we are excited to follow up on this, thank you for the suggestion.

d. To demonstrate the importance of phase separation in this association process, it would be nice to have a **McdB variant that does not phase separate but binds the carboxysome** shell (e.g. by inclusion of a solubilization tag such as MBP). This, however, may be a bit tricky to identify and validate.

   This is an excellent point. This was actually one of the major findings in our previous paper (Basalla et al., 2023, eLife). Here, we saw some phenotypes specifically related to the ability of McdB to phase separate, and have cited this in the discussion.

RE: Manuscript #E23-11-0443R
TITLE: "An invariant C-terminal tryptophan in McdB mediates its interaction and positioning function with carboxysomes"

Dear Dr. Vecchiarelli:

I am pleased to accept your manuscript for publication in Molecular Biology of the Cell.

Thanks for your exciting contribution to MBoC!

Sincerely,
Erin Goley
Monitoring Editor
Molecular Biology of the Cell

Dear Dr. Vecchiarelli:

Congratulations on the acceptance of your manuscript! Thank you for choosing to publish your work in Molecular Biology of the Cell (MBoC).

Within 10 days, an unedited PDF of your manuscript will be published on MBoC in Press, an early release version of the journal. The date your manuscript appears on this site is the official publication date.

Your copyedited and typeset manuscript will be scheduled for publication in the next available issue of MBoC. Our production team will notify you when the page proofs of your paper are ready for your review.

MBoC offers the option to publish your paper with immediate open access. Open access can increase the discoverability and usability of your research. If you would like to publish your paper with immediate open access but did not select this option during initial submission, please contact the MBoC Editorial Office (mbc@ascb.org).

We invite you to submit images related to your accepted manuscript to be considered for the journal cover.
Cover images must contain color, TIF Format
Size image at 7.4" wide x 5.85" deep
RGB and CMYK color (please submit two separate files, one in each format)
300 dpi resolution

Please also send a 1-paragraph description of the image that a general audience could understand. Include with the caption the name and institution of the individual(s) the image should be attributed to. Please provide this description in a Word file. You can send the file via email or by your favorite file transfer service to mboc@ascb.org.

Authors of Articles and Brief Communications are also encouraged to create a short video abstract to accompany their article when it is published. These video abstracts, known as Science Sketches, are up to 2 minutes long and will be published on YouTube and then embedded in the article abstract. Science Sketch Editors on the MBoC Editorial Board will guide you as you prepare your video. Information about how to prepare and submit a video abstract is available at www.molbiolcell.org/science-sketches. Please contact mboc@ascb.org if you are interested in creating a Science Sketch.

We look forward to publishing your paper in MBoC.
Sign up for MBoC's new content alerts here https://tinyurl.com/3bcsyk3e

Sincerely,

Eric Baker
Journal Production Manager
MBoC Editorial Office
mbc@ascb.org
